# Massive haemoptysis in rare congenital left-to-left shunt

**DOI:** 10.1093/ehjcr/ytae139

**Published:** 2024-03-15

**Authors:** Fabrizio Ricci, Kristian Galanti, Sabina Gallina, Cesare Mantini, Franco Fiore

**Affiliations:** Department of Neuroscience, Imaging and Clinical Sciences, ‘G. d’Annunzio’ University of Chieti-Pescara, Via dei Vestini 33, 66100 Chieti, Italy; Department of Clinical Sciences, Lund University, Jan Waldenströms gata 35, 221 00 Malmö, Sweden; University Cardiology Division, Heart Department, ‘SS Annunziata’ Polyclinic University Hospital, 66100 Chieti, Italy; Department of Neuroscience, Imaging and Clinical Sciences, ‘G. d’Annunzio’ University of Chieti-Pescara, Via dei Vestini 33, 66100 Chieti, Italy; Department of Neuroscience, Imaging and Clinical Sciences, ‘G. d’Annunzio’ University of Chieti-Pescara, Via dei Vestini 33, 66100 Chieti, Italy; Department of Neuroscience, Imaging and Clinical Sciences, ‘G. d’Annunzio’ University of Chieti-Pescara, Via dei Vestini 33, 66100 Chieti, Italy; Vascular Surgery Unit, Heart Department, ‘SS Annunziata’ Polyclinic University Hospital, 66100 Chieti, Italy

**Figure:**
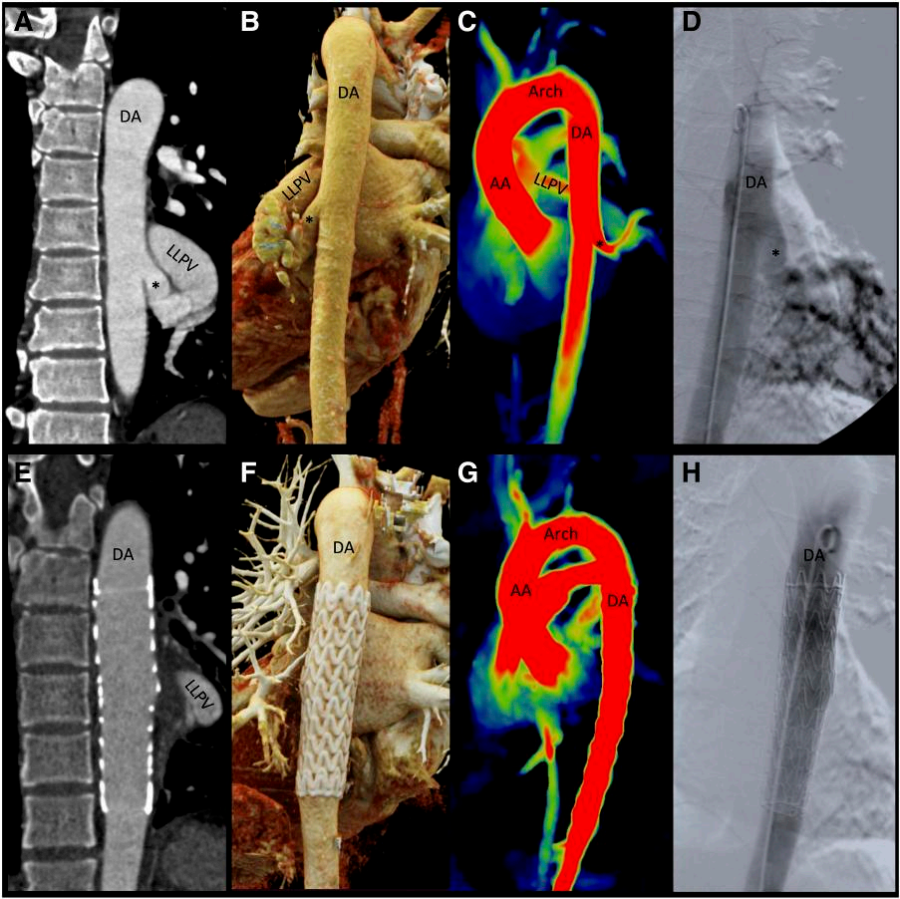


A 28-year-old man presented with recurrent haemoptysis. Chest computed tomography revealed the presence of a congenital descending aorta to pulmonary vein fistula (cDAPVF, asterisk, *Panels A* and *B*) with an aberrant vessel originating from the descending aorta and draining into a dilated left lower pulmonary vein (LLPV), diffuse alveolar haemorrhage characterized by ground glass opacities and consolidation, yet no parenchymal abnormalities to suggest sequestration (see Supplementary material online, *Video S1*). Echocardiography showed normal biventricular size and indices of systolic function. 4D flow magnetic resonance imaging highlighted the presence of a significant left-to-left shunt with an anterograde flow through the fistula of 35 mL (Qp/Qs = 0.6; *Panel C*; Supplementary material online, *Video S2*). The first-ever reported thoracic endovascular aortic repair (TEVAR) with coverage of the cDAPVF was successful (*Panels D* and *E*) in achieving swift resolution of vascular congestion (see Supplementary material online, *Video S1*) and normalization of haemodynamics (Qp/Qs = 1; *Panels F–H*; Supplementary material online, *Video S3*), with no recurrent haemoptysis at 1 year. Congenital descending aorta to pulmonary vein fistula is a rare vascular defect where aortic blood is shunted into the pulmonary venous circulation. Typically presenting in infancy with heart failure symptoms and haemoptysis, cDAPVF may remain asymptomatic until adulthood. Progressive shunting can lead to dilatation of pulmonary veins, left atrium, and pulmonary artery. Haemoptysis may occur due to rupture of small pulmonary veins. The case emphasizes the need for distinguishing cDAPVF from other vascular abnormalities, including pulmonary sequestration, scimitar syndrome, pulmonary varices, and arteriovenous malformations, as clinical implications and treatment can be profoundly different. It also highlights the role of multimodal imaging in supplying detailed anatomical and haemodynamic information for correct diagnosis and effective therapeutic guidance and introduces TEVAR as a viable treatment option for cDAPVF.

## Supplementary Material

ytae139_Supplementary_Data

## Data Availability

No data were generated or analysed for or in support of this paper.

